# Operational post-keratopasty graft tolerance due to differential HLAMatchmaker matching

**Published:** 2010-11-11

**Authors:** Daniel Böhringer, Frieder Daub, Johannes Schwartzkopff, Philip Maier, Florian Birnbaum, Rainer Sundmacher, Thomas Reinhard

**Affiliations:** University Eye Hospital Freiburg, Germany

## Abstract

**Purpose:**

Penetrating keratoplasty can restore vision in corneal blindness. However, immunologic rejection threatens graft survival. Matching donors at swine leukocyte antigen (SLA)-class II convey allo-specific tolerance in a large animal kidney-transplantation model despite mismatches at SLA-class I. The same matching pattern seems to account for the blood transfusion effect in kidney transplantation. Relying on the molecular basis of HLAMatchmaker eplets, we assessed whether this finding would also apply to keratoplasty, and if it would enhance the benefit from matching human leukocyte antigen (HLA)-class I alone.

**Methods:**

We retrospectively selected two independent cohorts comprising 586 and 975 penetrating keratoplasties. Our computations revealed a quantitative tolerogenicity factor analogous to the animal model. The number of mismatched HLA-class I eplets functioned as a factor for conventional histocompatibility. In the first cohort, we empirically determined the thresholds with the highest predictive power on graft rejection for both factors, and confirmed those thresholds in the second cohort. We applied Cox proportional hazards regression for these analyses.

**Results:**

The thresholds with highest predictive power revealed 220 eplets^2^ for the tolerance factor and 10 eplets for HLA-class I histocompatibility. The respective hazards ratios were 2.22 (p=0.04) versus 3.63 (p<0.01) in the first cohort and 2.09 (p<0.01) versus 1.51 (p=0.02) in the second, confirmatory cohort. The threshold factors proved to be additive in predicting immune reactions in both cohorts, (hazard ratios 2.66 in cohort 1 versus 1.70; p<0.01 in cohort 2).

**Conclusions:**

Operational tolerance may be inducible by balanced matching of HLA-class I and II HLAMatchmaker eplets. Furthermore, such tolerance is additive to histocompatibliity at HLA-class I.

## Introduction

Corneal diseases are among the five most common causes of blindness. Penetrating keratoplasty can restore vision in most cases. Yet a substantial percentage of grafts fail following immunologic rejection. Cumulative graft survival after five years is as low as 70% for all keratoplasty indications despite the widespread use of topical steroids [1].

Systemic immunoprophylaxis can improve overall graft survival in keratoplasty [2,3]. However, most ophthalmologists are reluctant to prescribe long-term systemic immunosuppressants because of potentially serious side effects.

This circumstance reinforces our need for effective primary prophylaxis of immunologic graft reactions. Graft rejection can be prevented employing graft-masquerade by human leukocyte antigen (HLA) matching, according to a recent report [4]. The controversial older literature in this field is usually biased due to low statistical power (too small cohorts) as well as the probably low accuracy of the HLA typings at that time [5]. HLA matching is, however, associated with prolonged waiting times depending on the individual HLA phenotype [6]. HLA matching is thus only routine nowadays in highly specialized transplantation centers.

Allo-specific tolerance is a related but distinct mechanism of long-term graft survival after withdrawal of all immunosuppressants. Graft rejection is prevented by alloantigen-specific immune regulation [7]. Consequently, donor-specific suppression of delayed-type hypersensitivity has been demonstrated in some long-term graft acceptors after kidney transplantation [8]. At least one major histocompatibility complex (MHC) locus must match between donor and recipient to induce allo-specific tolerance in the miniature swine model of kidney transplantation [9]. Good histocompatibility at the swine leukocyte antigen (SLA)-class II loci were considered inducive to tolerance in this large animal model, even when class I loci were loosely matched [10]. These findings resemble clinical observations in long-time survivors after kidney transplantation [8,11]. Additionally, the beneficial pre-blood transfusion effect is also ascribed to a loosely-matched HLA-class I loci in conjunction with closer agreement at HLA-class II [12].

On that basis, we hypothesized that matching at the HLA-DR locus more closely than at loci HLA-A and -B would induce graft tolerance in keratoplasty. However, conventional HLA-allele-based matching might be inappropriate for detecting this effect in the rather small HLA-typed cohorts available after keratoplasty. Moreover, the standard matching approach would not reflect structural or functional similarities between HLA alleles. HLAMatchmaker, by contrast, quantitatively assesses donor-recipient histocompatibility on the basis of polymorphic amino acid configurations (eplets) that represent structurally defined elements of the HLA epitopes [13]. HLAMatchmaker has already proved effective for quantitatively assessing HLA-class I histocompatibility in penetrating keratoplasty [14], and has recently been expanded to accommodate HLA-class II [15]. This new version enables us to test for the first time whether tolerance is induced from balanced histocompatibility at HLA-DR (class II) versus HLA-A and -B (class I).

## Methods

### Patients

We selected two independent cohorts of penetrating keratoplasties (Table 1). Complete HLA-A, -B, and -DR types were available for all donors and recipients in both groups.

**Table 1:** Two examples for calculating the tolerogenicity factor from the donor‘s and recipient‘s HLA-phenotypes.

**Examples**	**HLA class**	**Donor**	**Recipient**	**Eplet mismatches**	**Tolerance factor**
Example 1 (tolerogenic situation)	HLA-A/B	A*0301 A*2402 B*3501	A*0301 A*2402 B*4402 B*5601	**4**	7^2^–4^2^=**33**
	HLA-DR	DRB1*1101 DRB1*1501	DRB1*1101 DRB1*0401	7	
Example 2 (immunogenic situation)	HLA-A/B	A*0201 A*0301 B*4402	A*0201 A*2902 B*0702 B*4402	**4**	21^2^–4^2^=**425**
	HLA-DR	DRB1*0401 DRB1*1201	DRB1*0101 DRB1*1101	21	

Mismatched donor alleles are in boldface. The count of eplet mismatches was determined by HLAMachmaker. Eplet mismatches for HLA-A/B function as factor for conventional histocompatibility. The tolerance factor is calculated from the amount of mismatches from HLA-A/B and HLA-DR according to the equation shown in the Methods.

The first cohort of 586 patients was selected from the keratoplasty patients of the University Eye Hospital Düsseldorf, Germany. We included only keratoplasties without specific risk factors from the consecutive series of all corneal transplantations performed between 1995 and 2004. Indications were keratoconus, Fuchs endothelial dystrophy, bullous keratopathy and avascular corneal scars. Repeat keratoplasties were excluded. We selected this quite homogeneous subgroup to reduce confounders as much as possible. HLA types of donors and recipients at loci HLA-A, -B, and -DR were determined in a single laboratory accredited by the American Society for Histocompatibility and Immunogenetics [16]. Class I loci were typed serologically at low resolution; class II at high resolution using molecular methods.

The second cohort comprised 975 consecutive penetrating keratoplasties done at the University Eye Hospital Freiburg, Germany between 2003 and 2008. Complete HLA types of donors and recipients at loci HLA-A, -B, and -DR was the only inclusion criterion. This group thus included low-risk and high-risk patients (i.e., repeat keratoplasties, vascularized corneas and surface disorders). All HLA-types were determined in a single laboratory. Class I loci were typed at low, class II at high resolution, all using molecular methods.

### Penetrating keratoplasty and medical aftercare

All grafts were kept in organ culture for at least 6 days. We usually trephined with the Guided Trephine System (GTS; Polytech Ophthalmologie GmbH, Roßdorf, Germany) or modified Francescetti's trephines. One milligram fluocortolone per kilogram body mass was initially prescibed and tapered off within two weeks. Topical gentamycin 0.5% ointment was applied at least five times daily until complete re-epithelialization. Prednisolone-21-acetate 1% eye drops were administered five times daily during the first month, four times daily during the second, three times in the third month, twice in the fourth, and once in the fifth postoperative month. Most of the high-risk patients (second cohort only) were also given systemic cyclosporin and/or mycophenolate mofetil the for first year following keratoplasty [2].

### Assessment of immune reactions

The presence of endothelial precipitates, stromal edema or stromal infiltrates, and epithelial rejection lines were diagnosed as immune reactions. Routine visits were scheduled after 6 weeks and 4 and 12 months. Long-term follow-up took place annually.

### Statistical analyses

#### HLAMatchmaker assignments

We substituted the low resolution alleles with the high resolution alleles according to allele frequencies as described elsewhere [14]. We assigned HLAMatchmaker eplets to the HLA alleles based on HLAMatchmaker version 1.3. We considered only loci HLA-A, -B and -DR. Mismatched eplets (donor eplets absent in the recipient) were added up separately for HLA-classes I and II.

#### Computation of tolerance and histocompatibility factors

We created a function to quantify the tolerogenic situation from the mismatched HLAMatchmaker eplets according to our aforementioned hypothesis (equation, below). Low or negative values should predict the tolerogenic situation. This equation reveals that a well matched HLA-class II may be tolerogenic despite mismatches at HLA-class I [12]. We squared both addends to identify the highly mismatched constellations in particular.

$${\text{f}}_{\text{tolerogenic}}\left(\text{MI},\text{ MII}\right)={\left(\text{MII}\right)}^{\text{2}}-\;{\left(\text{MI}\right)}^{\text{2}}$$

MI symbolizes the number of mismatched HLAMatchmaker eplets at HLA-class I and MII the corresponding mismatches at HLA-class II.

We also added up the mismatched HLA-class I eplets separately. Two examples for these calculations are illustrated in Table 2. Histocompatibility at HLA-class I is a recognized risk factor for graft rejections after penetrating keratoplasty [14]. Our aim was to investigate the tolerogenic factor and HLA-class I histocompatibility factor as independent co-variates in the same Cox proportional hazards model. We counted only the immune reactions (reversible and irreversible) as endpoints. The factors' distributions in the cohorts are summarized in Table 3.

**Table 2:** Basic data of both cohorts.

**Factor**	**Cohort 1 (n=586)**	**Cohort 2 (n=975)**
Age at time of surgery (years)	59±20	58±19
Follow up (years)	2.8±2.2	1.7±1.2
Trephine diameter (mm)	7.8±0.3	8.0±0.4
Percentage of normal risk keratoplasties	100% (586)	36% (355)
**HLA-A/B Mismatches**		
0	3% ( 18)	4% (35)
1	11% (67)	24% (230)
2	25% (144)	28% (275)
3	40% (234)	27% (274)
4	21% (123)	17% (161)
**HLA-DR Mismatches**		
0	11% (62)	16% (152)
1	48% (284)	51% (505)
2	41% (240)	33% (318)

Years and millimeters are average ±standard deviation.

**Table 3:** Overview of the HLAMatchmaker-derived histotocompatibility assessments in both cohorts.

**Factor**	**Cohort 1 (n=586)**	**Cohort 2 (n=975)**
Eplet mismatches HLA-A/B	20±9	17±10
Eplet mismatches HLA-DR	14±7	13±8
Tolerogenic factor (see Equation 1)	−242±415	−182±408

Maximum of eplet mismatches are 49 and 33 at HLA-A/B versus HLA-DR. The tolerogenic factor ranges from −2176 to 1025.

### Threshold estimates and model validation

We assume that the effect of tolerogenicity and histocompatibility on immune reactions is nonlinear. We hypothesize that each factor must have a distinct threshold capable of identifying the beneficial constellations, as in HLAMatchmaker-matching before kidney transplantation [17]. In the first cohort, we empirically determined the respective thresholds with the highest predictive power on graft rejection for both factors. We chose the first (“low-risk”) cohort to achieve the highest level of statistical power during threshold estimation.

We generated two binomial factors based on the two thresholds for tolerance and HLA-class I histocompatiblity. These binomialized factors were then assessed in the second cohort for confirmation. The low versus high-risk classification was also fed into this confirmatory model to control for potential confounding risk factors for immunologic graft reactions.

## Results

### The first cohort: threshold determination

The thresholds with the highest predictive power possessed 220 eplets^2^ for the tolerance factor and 10 eplets for HLA-class I histocompatibility. The resulting hazards ratios regarding immune reactions were 2.22 for the tolerance factor and 3.63 for histocompatibility at HLA-class I loci A and B (Table 4). This means that the risk of immune reactions increases 2.22-fold in the patients with a tolerance factor >220 eplets^2^. The loosely HLA-class I-matched subgroup’s risk of immune reactions is 3.63 times higher than that of the more closely matched patients. Both factors proved to be additive in a third Cox proportional hazards model: the hazards ratio of both binomialized factors combined to a single factor by means of addition was also highly predictive for immune reactions (hazards ratio 2.66; p<0.01).

**Table 4:** Cox proportional hazards model with optimal thresholds for the tolerogenic factor and the HLAMatchmaker eplets at HLA-A/-B as fitted to the 586 normal-risk patients from cohort 1.

**Factor**	**Hazards ratio**	**p-value**
Tolerogenic factor higher than 220 eplets^2^	2.22	0.04
More than 10 HLAMatchmaker eplets at HLA-A/-B	3.63	<0.01

Immune reactions were the only endpoint.

### The second cohort: confirmatory analysis

**Figure 1:**
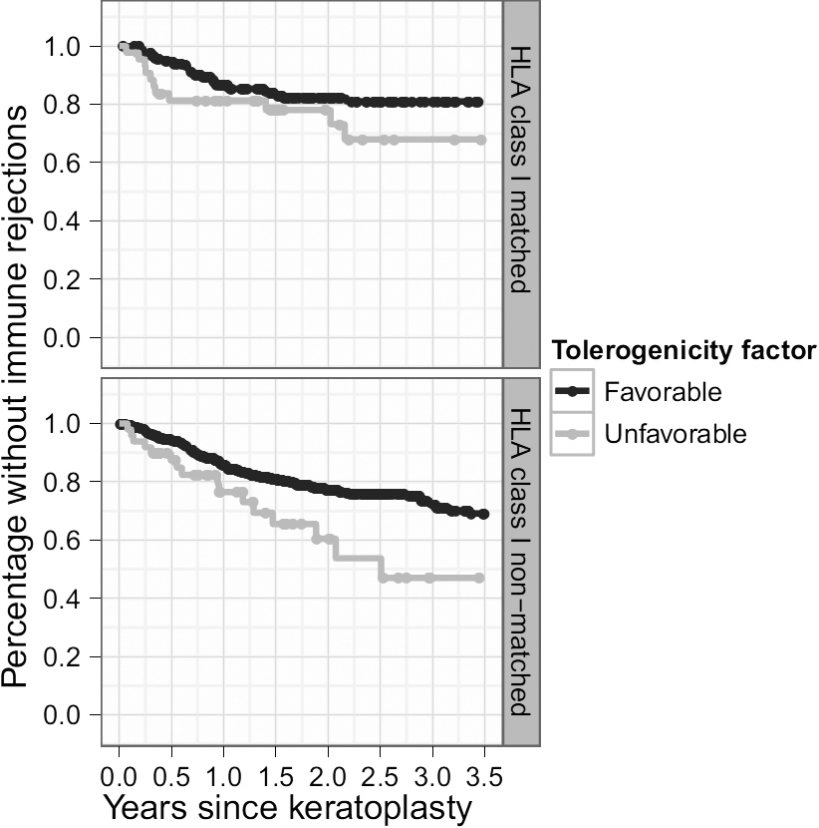
Kaplan–Meier estimations of rejection free survival for the 975 patients in cohort 2. The HLA-class I matched (less or equal 10 mismatched HLAMatchmaker eplets) and non-matched (>10 mismatched HLAMatchmaker eplets) sub-groups are displayed separately. More immune reactions occur when tolerance factor higher than 220 eplets^2^ (light gray) in comparison to keratoplasties with the tolerance factor lower or equal than 220 eplets^2^ (dark gray). This difference seems to be even more pronounced in the non-HLA-class I matched subgroup.

Both the binomialized tolerance factor and binomialized HLA-class I histocompatibility also predicted immune reactions in the second (confirmatory) cohort (Table 5). Again, both factors proved to be additive in a separate Cox proportional hazards model (hazards ratio 1.70; p<0.01). The corresponding Kaplan–Meier curve appears in Figure 1, illustrating our findings’ clinical benefit.

**Table 5:** Validation of the thresholds for HLA-class I histocompatibility and the tolerance induction from Table 4.

**Factor**	**Hazards ratio**	**p-value**
Tolerogenic factor higher than 220 eplets^2^	2.00	<0.01
More than 10 HLAMatchmaker eplets at HLA-A/-B	1.50	0.02
Risk estimation at time of keratoplasty (low versus high risk)	0.56	<0.01

This Cox model is fitted to cohort 2 comprising of 975 consecutive patients. The model is adjusted for low- versus high-risk keratoplasties. Immune reactions were the only endpoint.

## Discussion

Our findings clearly indicate that matching for HLA-class I using the HLAMatchmaker can prevent immune reactions in normal- and high-risk keratoplasty. This finding has been previously reported [14] and is in line with other reports on the conventional HLA-matching effect in keratoplasty [18-20].

Paradoxically, matching at HLA-DR can further reduce the risk of immune reactions when HLA-class I is only loosely matched. We herein ascribe this unexpected finding to the induction of operational tolerance. Our interpretation is guided by the circumstance that close matches at SLA-class II induce allo-specific tolerance in a large animal model of kidney transplantation, despite mismatches at SLA-class I [9]. This HLA constellation also seems to prolong kidney graft survival in the clinical setting [8,11]. Moreover, the immune-modulating effect of HLA-DR-shared allogeneic blood transfusions on the allo-immune response after kidney transplantation (”pre-transfusion-effect”) has been linked to tolerance induction [12].

We speculate that on the cellular level, donor-derived antigen-presenting cells (APCs) expose transplantation antigens that are derived from mismatched HLA-class I epitopes. These APCs can be modulated by IL-10 to specifically promote a regulatory immune response toward exposed HLA-class I-derived antigens [21]. Recent findings indicate that IL-10 may in fact be upregulated in post-keratoplasty eyes [22]. Such modulated APCs may give rise to allo-specific CD4+ regulatory T-cells (Tregs) provided donor and recipient share enough HLA-DR-derived eplets. These Tregs may eventually permanently down-regulate the allo-immune response toward the organ donor [23].

Interestingly, HLA-DR matching is controversial in keratoplasty. Concordance at the HLA-DR locus has even been suspected of reducing graft survival in a meta analysis [24]. However, DR-locus matching proved beneficial in a large monocentric keratoplasty cohort [18]. This paradox was ascribed to presumed HLA-typing errors in the older investigations [18]. An alternative interpretation may result from the interaction with HLA-class I histocompatibility that we are proposing. According to the equation presented above (Methods), unfavorable constellations with respect to tolerance induction can occur when the HLA-class I and HLA-class II loci are not matched very closely.

While HLAMatchmaker was designed with antibody epitopes in mind, we presume that the concordance at the HLAMatchmaker eplet level also concurs with T-cell reactivity to some extent. This assumption is strengthened by our analysis of HLA-class I histocompatibility, although it is based on the older ('triplet') version of HLAMatchmaker [14]. We are aware of no other investigation on the newer HLAMatchmaker class I/class II eplets in this context.

The statistical power in our investigation was reduced because all the HLA-class I types had been performed at low resolution. Our approach was to select the most likely four-digit HLA-alleles based on German allele frequencies. This approach inevitably resulted in ambiguities when assigning the HLAMatchmaker eplets. The size of the actual effect from our approach (Figure 1) would thus probably have been higher based on high resolution typing.

Striving for utmost coincidence between donor and recipient is associated with waiting times over a year in most situations [6]. Herein we describe a method than can identify a small subset of donor-recipient combinations especially at risk of immune reactions. This subset is identified from unfavorable donor-recipient HLA constellations with respect to tolerogenicity, as well as HLA-class I histocompatibility. Thinking translationally, it would be feasible to prospectively and systematically avoid these rather rare graft-recipient constellations in the clinical routine. The additional benefit from actively seeking HLA class I compatible and tolerogeneic donors could be reserved for high-risk patients and/or discussed with the patients individually.

In summary, our findings suggest a substantial benefit from the systematic typing of all donors and patients, preferably with resolution at four-digit alleles. On this basis, long-term prognosis in keratoplasty may be improved in the future.
